# Perturbation of the human gastrointestinal tract microbial ecosystem by oral drugs to treat chronic disease results in a spectrum of individual specific patterns of extinction and persistence of dominant microbial strains

**DOI:** 10.1371/journal.pone.0242021

**Published:** 2020-12-01

**Authors:** Hyunmin Koo, Casey D. Morrow

**Affiliations:** 1 Department of Genetics, University of Alabama at Birmingham, Birmingham, Alabama, United States of America; 2 Department of Cell, Developmental and Integrative Biology, University of Alabama at Birmingham, Birmingham, Alabama, United States of America; McMaster University, CANADA

## Abstract

**Background:**

Oral drugs can have side effects such as diarrhea that indicate the perturbation of the gut microbial community. To further understand the dynamics of perturbation, we have assessed the strain relatedness of samples from previously published data sets from pre and post bowel evacuation, episodes of diarrhea, and administration of oral drugs to treat diabetes and rheumatoid arthritis.

**Methods:**

We analyzed a total of published five data sets using our strain-tracking tool called Window-based Single Nucleotide Variant (SNV) Similarity (WSS) to identify related strains from the same individual.

**Results:**

Strain-tracking analysis using the first data set from 8 individuals pre and 21–50 days post iso-osmotic bowel wash revealed almost all microbial strains were related in an individual between pre and post samples. Similarly, in a second study, strain-tracking analysis of 4 individuals pre and post sporadic diarrhea revealed the majority of strains were related over time (up to 44 weeks). In contrast, the analysis of a third data set from 22 individuals pre and post 3-day exposure of oral metformin revealed that no individuals had a related strain. In a fourth study, the data set taken at 2 and 4 months from 38 individuals on placebo or metformin revealed individual specific sharing of pre and post strains. Finally, the data set from 18 individuals with rheumatoid arthritis given disease-modifying antirheumatic drugs methotrexate or glycosides of the traditional Chinese medicinal component *Tripterygium wilfordii* showed individual specific sharing of pre and post strains up to 16 months.

**Conclusion:**

Oral drugs used to treat chronic disease can result in individual specific microbial strain change for the majority of species. Since the gut community provides essential functions for the host, our study supports personalized monitoring to assess the status of the dominant microbial strains after initiation of oral drugs to treat chronic disease.

## Introduction

One of the features of the healthy gut microbiome is a stable microbial community that has the resiliency to recover following perturbation to maintain essential functions such as colonization resistance and host cell metabolism [1, 2]. The capacity for recovery of the microbial community is most evident from studies that have examined the impact of the antibiotics on the gastrointestinal (GI) tract microbial communities wherein most of the time the community structure of normal adults recovers after single and even multiple-dose antibiotic treatments [3–8]. The resiliency of the gut microbial community to recover from perturbation is also evident following the loss of biomass after designed evacuation (*i*.*e*. bowel wash) or studies that have shown disruption and recovery of the gut microbial community following biomass evacuation resulting from disease-related diarrhea [9–14].

Non-antibiotic medications, including oral drugs used for the treatment of chronic diseases, have been found to impact the composition of the gut microbial community [15]. Notably, metformin, one of the most widely prescribed drugs for the treatment of type 2 diabetes (T2D), is known to have side effects of diarrhea and flatulence, which can be indicative of a disruption of the gut microbial community structure [16–22]. Previous studies have also suggested a link between gut microbial community disruption and anti-rheumatic drugs [23, 24]. Oral methotrexate is one of the most commonly used anti-rheumatic drugs and more recently glycosides of the traditional Chinese medicinal component *Tripterygium wilfordii* (thunder god vine (T2)) have known side effects of diarrhea consistent with disruption of the gut microbial community [25, 26].

Previous studies have relied on a targeted metagenomic sequencing method such as 16S rRNA sequencing to determine the relative abundance of microbes as a means to assess the gut microbial community structure and their functions [1, 27]. However, by using a shotgun metagenomic sequencing approach in conjunction with the in-depth strain-tracking analysis, the emergence of new microbial strains was able to be examined in the gut microbial ecosystem [28–30]. In our previous studies, we have applied our strain-tracking tool, called a Window-based Single Nucleotide Variant (SNV) Similarity (WSS), on metagenomic sequencing data sets to assess the strain relatedness for each individual over time, supporting the concept of a microbiome fingerprint [28, 31–36]. Each species’ cut-off values that can discern between related and unrelated individual pairs were established based on the Human Microbiome Project (HMP). Using WSS analysis, we previously have demonstrated the emergence of new microbial strains following antibiotics or physical alteration of the GI tract environment [33, 34]. We have also used this analysis to demonstrate the mother to infant microbial strain transmission and to determine that gut microbial strains are stable in some humans for up to decades [35, 36].

In this study, we have expanded our strain-tracking analysis to investigate whether new strains emerge after non-antibiotic perturbation of the gut microbial community. To do this, we have made use of existing metagenomic data sets from fecal samples from individuals given an iso-osmotic agent or individuals with sporadic diarrhea. We also compared the impact on the GI tract strain composition with long-term oral drugs used for diabetes (metformin) or anti-rheumatic drugs methotrexate (*herein* MTX) and glycosides of the traditional Chinese medicinal component *Tripterygium wilfordii* (thunder god vine; *herein* T2). The results of our strain-tracking analysis provide a new perspective that while the gut microbial strain profile is not impacted by bowel evacuation or mild diarrhea, there is an individual specific recovery pattern of the microbial strain profile following long term oral drugs that could influence the known functions of the gut microbial community in the health of the host.

## Materials and methods

### Public data sets

In this study, we used publicly available data sets for individuals undergoing iso-osmotic bowel wash (Fukuyama et al.) [10] and sporadic diarrhea (Lloyd-Price et al.) [37]. For Fukuyama et al., fecal samples from 8 healthy individuals were collected approximately 10 weeks before and 10 weeks after mechanical bowel wash [10]. Briefly, on the morning of the bowel wash, 8 participants were instructed to drink 300 mL of a solution (*GoLytely*) containing polyethylene glycol (PEG) and electrolytes every 10 minutes (up to 4L total) until their diarrhea was clear and watery. For the Lloyd-Price et al., a subset of 4 individuals who had a diarrhea(s) (but did not diagnose with inflammatory bowel diseases (IBD) or recent treatment with antibiotics) were selected to run further analysis [37]. We also used the two studies that described the impact of metformin for individuals with newly diagnosed T2D (1) treated with metformin for 3 days (Sun et al. [21]) and (2) treated with placebo or metformin for 2 and 4 months (Wu et al. [38]). For the Sun et al., fecal samples were taken from 22 individuals with T2D at pre and 3 days post metformin treatment (1,000 mg twice daily for 3 days) [21]. For the Wu et al., fecal samples were collected from a total of 38 individuals treated with either placebo or metformin (1700mg/day) at pre, 2 months and 4 months [38]. In addition, we used the Zhang et al. data set that described the impact of anti-rheumatic drugs for individuals with rheumatoid arthritis (RA) [26]. Fecal samples collected from 18 individuals at pre and 3–16 months post anti-rheumatic drugs treatment either MTX or T2 [26]. All data set used in this study was summarized in **S1 Table**.

### Sequence reads and processing

A total of 6,540,858,979 metagenomic sequencing reads were downloaded from the five public data sets; 931,584,161 reads from the Fukuyama et al., 199,476,279 from the Lloyd-Price et al., 1,284,404,173 from the Sun et al., 2,939,361,954 from the Wu et al., and 1,186,032,412 from the Zhang et al. (**S2 Table**). Sequences reads were filtered to remove short sequences (sequence length <50 bases), low quality reads (sliding window of 50 bases having a QScore <20) using Trimmomatic (version 0.36) [39], and any human reference genome (hg19) using bowtie2 (version 2.3.4.3) with default parameters [40]. The quality processed reads were then used for the downstream analyses (**S2 Table**).

### Strain-tracking analysis using WSS

To investigate strain relatedness for each individual between pre and post bowel wash samples from Fukuyama et al. data set [10], we have merged all pre bowel wash samples into a single sample, and also combined multiple post bowel wash samples into two separate time points, post 1–10 days and post 21–50 days. The strain-tracking analysis also applied for the Lloyd-Price et al. data set [37] to examine strain relatedness for each individual over time by comparing samples that collected at the beginning of the experiment to the samples collected post weeks ranges from 27 to 44. From the Sun et al. data set [21]., the strain-tracking analysis was conducted for each individual by comparing pre samples to post 3-day metformin treated samples. From the Wu et al. data set [38], strain relatedness of individuals when metformin treated with the extended time point up to 4 months was examined using WSS analysis by comparing pre samples to each of post samples collected at 2- and 4-month. Similarly, to better understand the impact of metformin on the microbial community, individuals from the placebo group were selected from the Wu et al. data set [38] and used for strain-tracking analysis. Strain relatedness of individuals from the placebo group was examined by comparing pre samples to each of the post samples collected at 2- and 4-month. From Zhang et al. [26], the strain-tracking analysis was performed for each individual by comparing pre and 3–16 months post anti-rheumatic drugs (either MTX or T2) treatment samples.

For the WSS analysis, the processed reads were aligned to the 93 reference sequences, which were previously constructed based on the HMP data set [28, 32, 34, 35] using the Burrows-Wheeler aligner program (BWA; version 0.7.13) [41]. For each individual, multi-sample SNVs for each given reference sequence were measured among all samples using the Genome Analysis Toolkit (GATK; version 3.7) [42]. The resultant Variant Call Format (VCF) files were then used for pairwise comparisons between every possible pair of samples for the individual to measure their overall genome-wide SNV similarity for each microbial species. Any samples with a low sequence coverage (<30%) and depth (<3.5) against their given reference sequences were excluded from the pairwise comparisons [28, 36]. Also, low coverage windows with more than 50% of the bases having a read depth < 5 were also excluded to compare SNV similarity between sample pairs. After all the filtering processes, species that were able to provide the WSS score were only selected from each data set. To distinguish a related strain pair at various time points for each individual, the resultant WSS score for each species was compared against each species’ cut-off value (Related strain pair: WSS score > cut-off; Unrelated strain pair: WSS score < cut-off) [34, 35]. The resultant comparison analysis was then summarized and visualized using the ggplot2 package (version 3.3.0) (https://cran.r-project.org/web/packages/ggplot2/index.html) in R (version 3.5.1) software [43]. Species that did not have a cut-off value were excluded from the analysis. All codes implemented in the WSS were previously deposited and are available at https://github.com/hkoo87/mgSNP_2.

Strain-tracking analysis for *Bacteroides vulgatus* was additionally performed for bowel wash (Fukuyama et al. [10]), metformin for 3 days (Sun et al. [21]), metformin or placebo for 2 and 4 months (Wu et al. [38]), and anti-rheumatic drugs (MTX or T2) (Zhang et al. [26]) data sets using StrainPhlAn with default parameters [30]. Aligned sequence reads against the set of species-specific marker gene database implemented in MetaPhlAn [44, 45] were used to build a phylogenetic tree of the strains [30]. The resultant tree for *B*. *vulgatus* was then visualized using neighbor-joining method along with the Maximum Composite Likelihood [46] in MEGA X [47, 48].

## Results

### Strain-tracking of individuals post bowel wash or diarrhea

**Fig 1:**
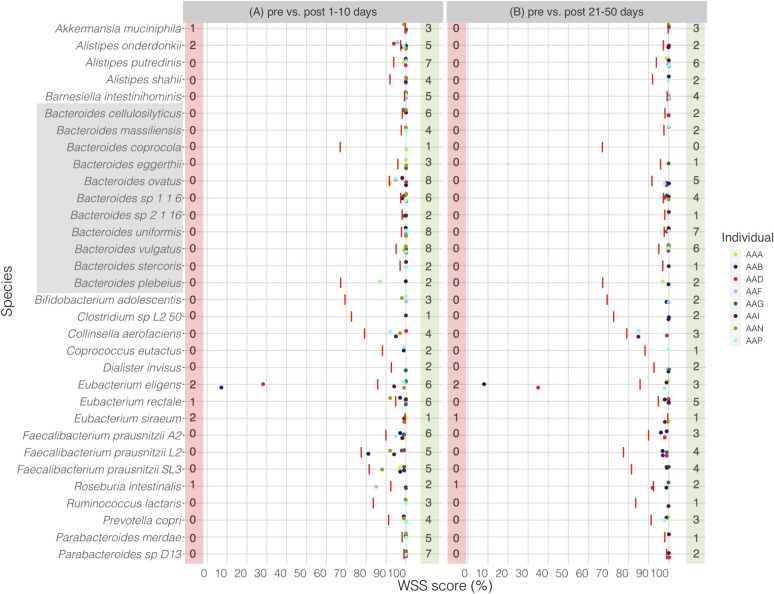
WSS analysis for the data set from Fukuyama et al.

Fukuyama et al. described the impact of iso-osmotic disruption of the human GI tract [10]. We compared the microbial strains of the pre samples with those strains found at two post time point samples: 1–10 days (early time points) and 21–50 days (later time points). We found that the majority of the pre strains were recovered in nearly all individuals by post 1–10 days (earliest normal bowel movements **Fig 1A**). However, for some individuals, *Akkermansia muciniphila*, *Alistipes onderdonkii*, *Eubacterium eligens*, *Eubacterium rectale*, *Eubacterium siraeum* and *Roseburia intestinalis* were found to have unrelated strain between pre and post samples (**Fig 1A**). Although the unrelated strain was still observed in some species, including *E*. *eligens*, *E*. *siraeum*, *and R*. *intestinalis* for a few individuals, the remaining species had the pre strain by post 21–50 days (**Fig 1B**). All pairwise comparisons conducted between pre and post samples are shown in the **S3 Table**. We have additionally performed StrainPhlAn analysis for *B*. *vulgatus* across all samples to assess strain relatedness between samples. We found a match for 4 of 6 cases between WSS and StrainPhlAn, representing a small difference (<0.001) in branch length (**S1 Fig**). Overall, these results highlight the recovery of the GI tract microbial communities following the common bowel wash procedure with the persistence of the majority of the species, particularly *Bacteroides spp*. after up to 50 days of the disruption.

The resultant WSS scores of 8 individuals including each species’ cut-off value (red line) are shown in a scatter plot by using ggplot2 package in R software. All pairwise comparisons were conducted for individuals between (A) pre bowel evacuation vs. post 1–10 days bowel evacuation, and (B) pre bowel evacuation vs. post 21–50 days bowel evacuation. The red shaded box includes a number of individuals who had unrelated strain pair between pre and post bowel evacuation samples, and the green shaded box includes a number of individuals who had related strain pairs. The gray shaded box includes all *Bacteroides spp*.

**Fig 2:**
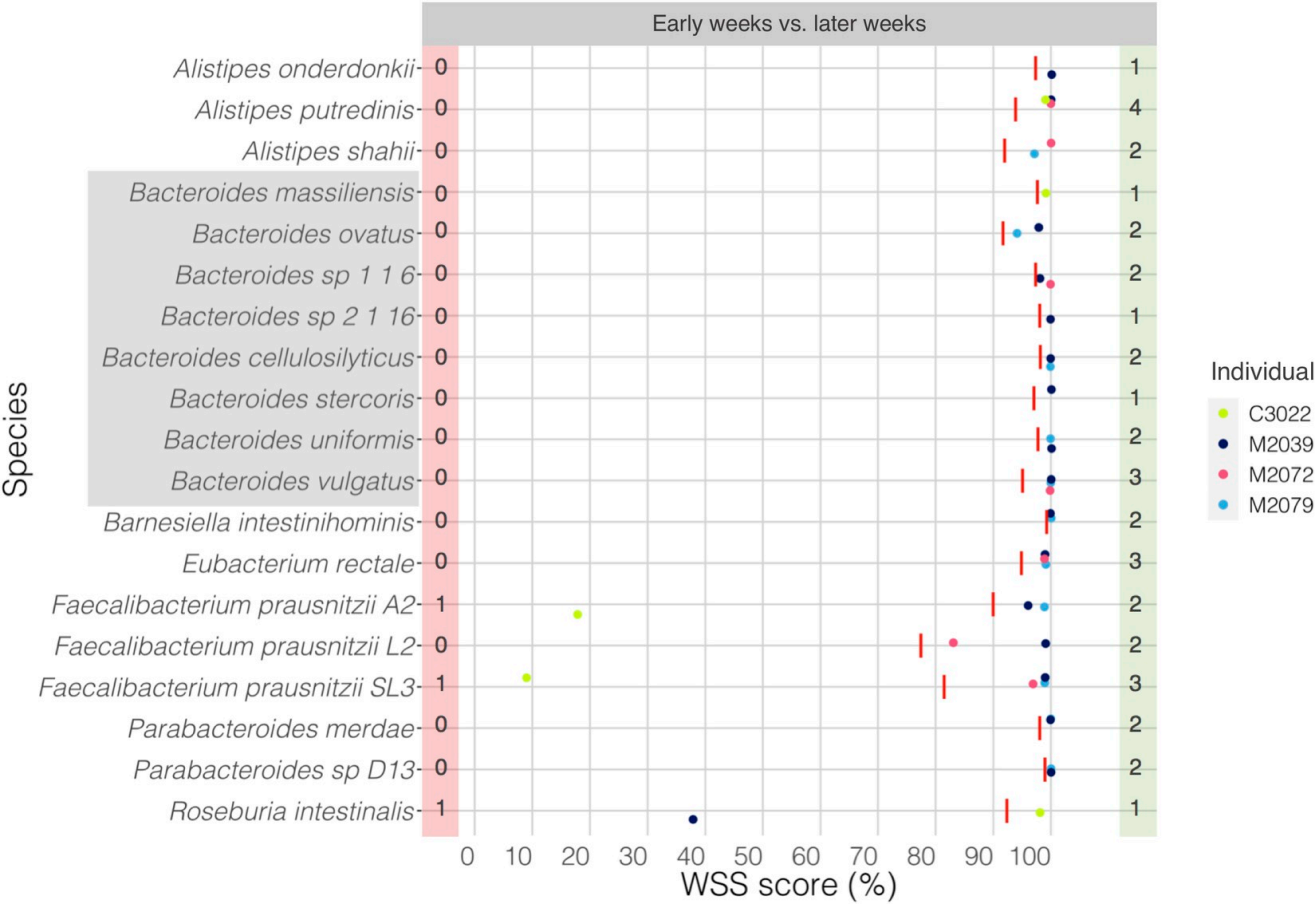
WSS analysis for the data set from Lloyd-Price et al.

Next, we have analyzed 4 individuals from Lloyd-Price et al. data set [37]. These individuals were classified as “non-IBD” according to Lloyd-Price et al. and selected for their study based on their initial endoscopic and histopathologic findings [37]. We have selected a subset (*n* = 4) from the non-IBD group to run strain-tracking analysis based on individuals who had 1–2 episodes of diarrhea during the collection time and were in the age range with the absence of antibiotics time of sample collection similar to that for the other samples in our study (**Fig 2).** The resultant of the strain-tracking analysis showed that the majority of the species, particularly *Bacteroides spp*. had the related strain over time, ranges from 27 to 44 weeks. One individual (M2039) had an unrelated strain of *R*. *intestinalis* over time, and another individual (C3022) had an unrelated strain of *Faecalibacterium prausnitzii A2* and *Faecalibacterium prausnitzii SL3* (**Fig 2**). All pairwise comparisons conducted for individuals over time are shown in the **S4 Table**.

The resultant WSS scores of 4 individuals including each species’ cut-off value (red line) are represented in a scatter plot by using ggplot2 package in R software. All pairwise comparisons were conducted for individuals between early weeks vs. later weeks. The red shaded box includes a number of individuals who had unrelated strain pair over time, and the green shaded box includes a number of individuals who had related strain pair. The gray shaded box includes all *Bacteroides spp*.

### Strain-tracking of individuals post metformin treatment

**Fig 3:**
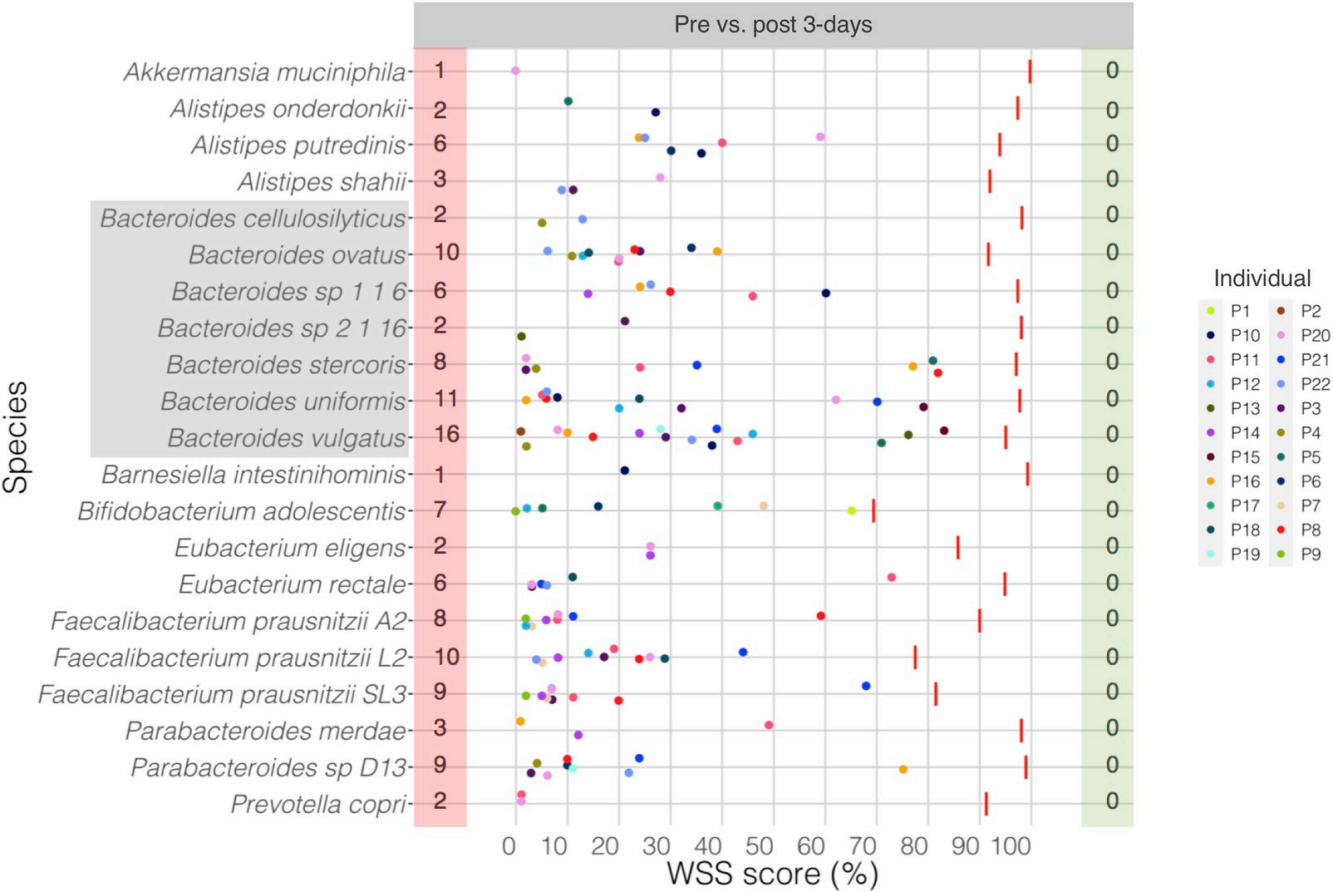
WSS analysis for the data set from Sun et al.

Sun et al. had shown disruption of the composition of the microbial community following a 3-day treatment with metformin [21]. Consistent with this result, our analysis showed a massive disruption of the microbial strains for all compared species with all individuals between pre and 3-day post treatment samples (**Fig 3**). This result demonstrates a complete disruption in the GI strain composition following the initiation of metformin. All pairwise comparisons conducted between pre and post 3-day metformin treated samples are shown in the **S5 Table**. Additional StrainPhlAn analysis for *B*. *vulgatus* across all samples represented 14 of 22 cases matched between WSS and StrainPhlAn (**S2 Fig**).

The resultant WSS scores of 22 individuals including each species’ cut-off value (red line) are represented in a scatter plot by using ggplot2 package in R software. All pairwise comparisons were performed for individuals between pre and post 3-day metformin treatment. The red shaded box includes a number of individuals who had unrelated strain pair between pre and post 3-day metformin treatment, and the green shaded box includes a number of individuals who had related strain pair. The gray shaded box includes all *Bacteroides spp*.

**Fig 4:**
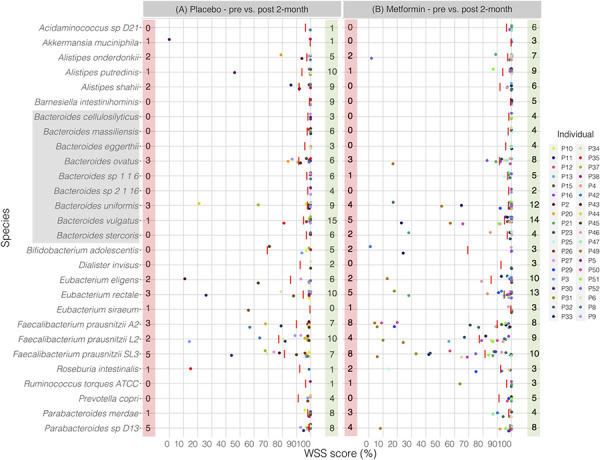
WSS analysis for the 2-month treatment data set from Wu et al.

**Fig 5:**
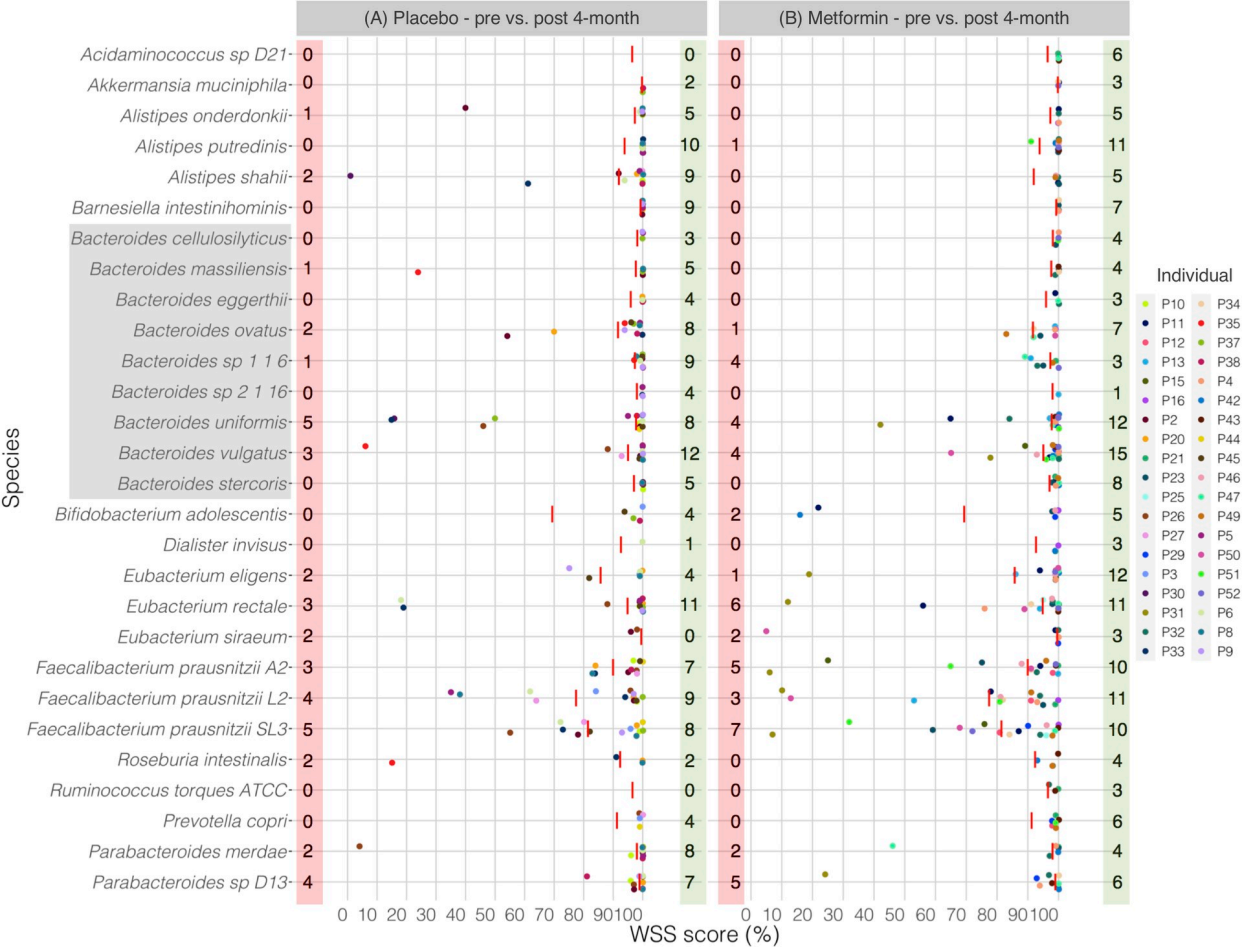
WSS analysis for the 4-month treatment data set from Wu et al.

Next, we wanted to determine if the effects of metformin on the strain composition would be detected after an extended time of using metformin. To do this, the data set from Wu et al. was analyzed to examine the strain composition of the gut microbial community 2 and 4 months post initiation of metformin [38]. In addition, each individual from the placebo-controlled group was separately compared between pre and 2- and 4-month post samples. Comparison of the pre sample with the 2-month post placebo treatment sample revealed unrelated strains including members of genera *Akkermansia*, *Alistipes*, *Bacteroides*, *Eubacterium*, *Faecalibacterium*, *Roseburia*, and *Parabacteroides* (**Fig 4A**). Interestingly, a similar result was found between pre and 2-month post metformin treatment showing several unrelated strains including members of genera *Alistipes*, *Bacteroides*, *Bifidobacterium*, *Eubacterium*, *Faecalibacterium*, *Roseburia*, *Ruminococcus*, and *Parabacteroides* (**Fig 4B**). Even at 4-month after treated with either placebo or metformin, most of the individuals still had unrelated strains including members of genera *Alistipes*, *Bacteroides*, *Bifidobacterium*, *Eubacterium*, *Faecalibacterium*, *Roseburia*, and *Parabacteroides* between pre and post samples (**Fig 5A and 5B**). All pairwise comparisons conducted between pre and post 2- and 4-month metformin treated samples are shown in the **S6 Table**. We have additionally conducted StrainPhlAn analysis for *B*. *vulgatus* across all samples. From this analysis, we found 12 of 14 cases were agreed for placebo treatment and 16 of 20 cases matched for metformin treatment between WSS and StrainPhlAn (**S3 Fig**).

Combining the information from both metformin data sets, a picture for the impact of metformin in the gut microbial strain composition emerges that after initiation of metformin in T2D patients a drastic disruption of the gut microbial strain community occurs for a short time (at least 3 days) with the eventual recovery of the microbial strain composition by 2 and 4 months to be similar to the pre metformin composition (**S7 Table**)

The resultant WSS scores of 38 individuals including each species’ cut-off value (red line) are displayed in a scatter plot by using ggplot2 package in R software. All pairwise comparisons were conducted for individuals between (A) pre sample vs. post 2-month placebo treatment, and (B) pre sample vs. post 2-month metformin treatment. The red shaded box includes a number of individuals who had unrelated strain pair between pre and post 2-month treatment, and the green shaded box includes a number of individuals who had related strain pair. The gray shaded box includes all *Bacteroides spp*.

The resultant WSS scores of 38 individuals including each species’ cut-off value (red line) are showed in a scatter plot by using ggplot2 package in R software. All pairwise comparisons were performed for individuals between (A) pre sample vs. post 4-month placebo treatment, and (B) pre sample vs. post 4-month metformin treatment. The red shaded box includes a number of individuals who had unrelated strain pair between pre and post 4-month treatment, and the green shaded box includes a number of individuals who had related strain pair. The gray shaded box includes all *Bacteroides spp*.

### Strain-tracking of individuals post anti-rheumatic drugs

**Fig 6:**
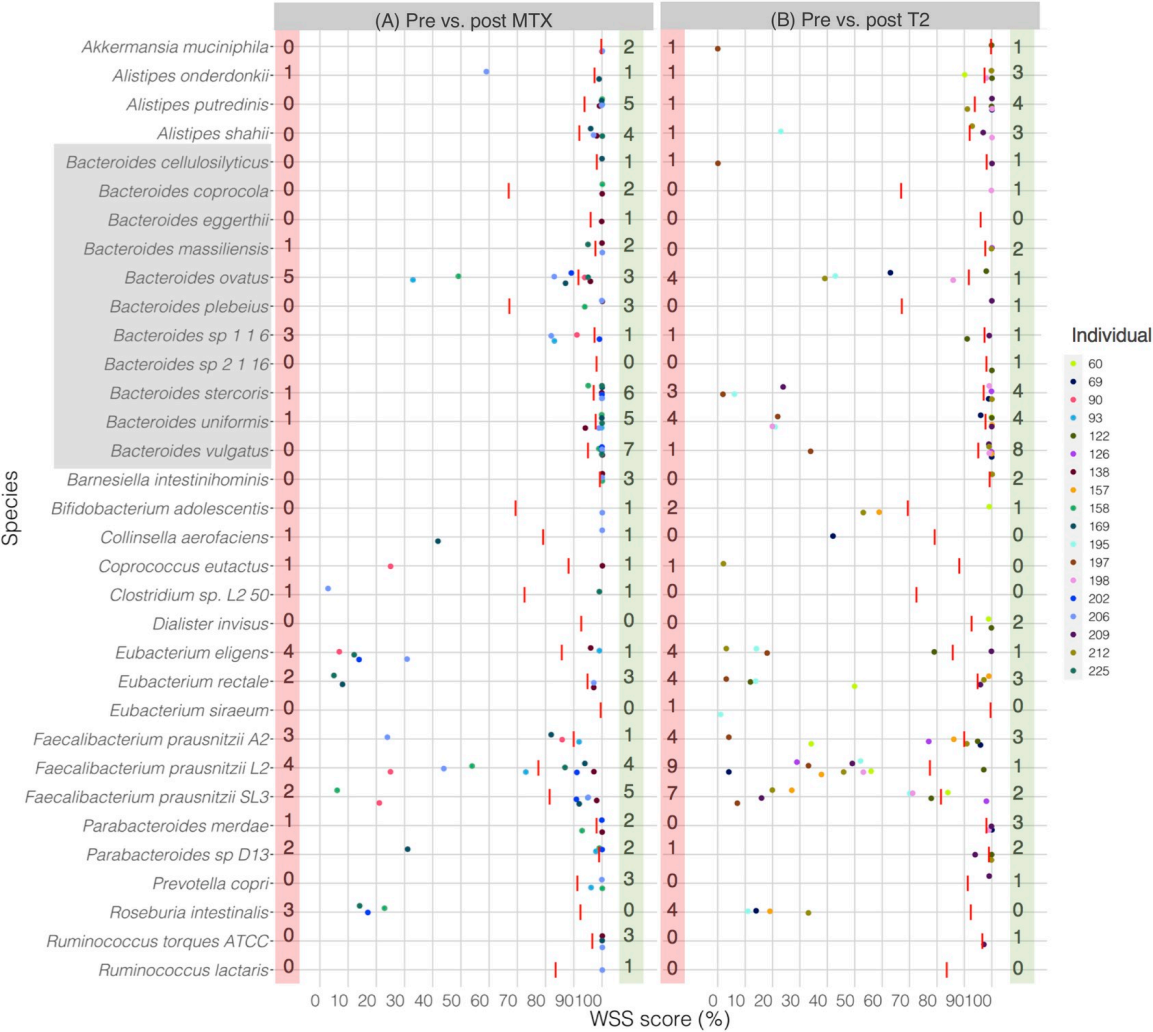
WSS analysis for the data set from Zhang et al.

In a recent study, Zhang et al. showed that the gut microbiome was disrupted in RA patients that have been treated with oral disease modifying drugs such as MTX or T2 [26]. We have analyzed this data set to see the impact of these individual oral drugs on the strain stability of the gut microbes (**Fig 6**). We found that individual specific response with respect to the gut microbial strain stability post MTX and T2 treatment. Comparison of the pre sample with the post MTX treatment sample showed unrelated strains including members of genera *Alistipes*, *Bacteroides*, *Collinsella*, *Coprococcus*, *Clostridium*, *Eubacterium*, *Faecalibacterium*, *Parabacteroides*, and *Roseburia* (**Fig 6A**). Similarly, unrelated strains including members of *Akkermansia*, *Alistipes*, *Bacteroides*, *Bifidobacterium*, *Coprococcus*, *Eubacterium*, *Faecalibacterium*, *Parabacteroides*, and *Roseburia* were observed between pre and post T2 treatment (**Fig 6B**). All pairwise comparisons conducted between pre and post MTX or T2 treated samples are shown in the **S8 Table**. Additional analysis from StrainPhlAn showed that there were 13 of 20 cases matched between WSS and StrainPhlAn (**S4 Fig**).

The resultant WSS scores of 18 individuals including each species’ cut-off value (red line) are represented in a scatter plot by using ggplot2 package in R software. All pairwise comparisons were performed for individuals between (A) pre sample vs. post MTX treatment, and (B) pre sample vs. post T2 treatment. The red shaded box includes a number of individuals who had unrelated strain pair between pre and post treatment, and the green shaded box includes a number of individuals who had related strain pair. The gray shaded box includes all *Bacteroides spp*.

## Discussion

In this study, we examined the persistence and extinction of microbial strain followed by various perturbations on the GI tract. We first demonstrated that the majority of the microbial strains, particularly for *Bacteroides spp*., were persistent over time in individuals after bowel evacuation using iso-osmotic disruption and sporadic diarrhea. In contrast, the oral drug, metformin, caused extensive microbial strain change for the first three days in individuals. The strain change was still observed for certain species in individuals by 2- and 4-months post metformin treatment although more pre-strains were recovered by 4 months. Disease-modifying anti-rheumatic drugs, MTX or T2, also caused microbial strain change for certain species in individuals post treatment. Comparing appearance of unrelated strains post oral drug treatments showed an oral drug specific strain change for each individual (**S9 Table**). Collectively, our results from these studies demonstrate an individual specific impact of different oral drugs on the dominant microbial strains in the GI ecosystem.

Understanding the dynamics of recovery of the gut microbiome community after a disruption is important because of the important role of the gut ecosystem in the overall health of the host. While studies using the targeted metagenomic sequencing approach have provided substantial information on the gut microbial community structure, the analysis does not provide sufficient resolution for the identification of microbes at the level of species or strains. The use of high-density sampling coupled with shotgun metagenomic sequencing has provided data sets such as those used in the current study that can be used to further elucidate the impact of disruptions on the microbial ecosystem. In our previous studies using strain-tracking WSS analysis, we showed a capacity to recover dominant strains followed by GI tract disruptions such as antibiotic treatment and surgical procedures [33, 34]. Consistent with these results, we found that individuals that had undergone bowel evacuation either by mechanical disruption or sporadic diarrhea recovered with nearly the complete spectrum of the pre disruption strains. Collectively, our results establish that bowel evacuation and sporadic diarrhea *per se* does not impact the recovery of the dominant microbial strains. Indeed, this would be consistent with the idea that these bowel evacuations (*e*.*g*. defecation) and sporadic diarrhea more closely represent the normal physiological processes that do not disrupt the microbial strain composition within the niches of the GI tract [49].

In contrast, we found evidence for the appearance of new microbial strains post administration of oral drugs used for the treatment of chronic disease. Sun et al. demonstrated a uniformly altered gut microbial community composition followed by the 3-day administration of metformin on 22 individuals [21]. Using their metagenomic data set of Sun et al, we found the 3-day administration of metformin provided the most dramatic example of the disruption of the microbial strains in the gut ecosystem. From our strain-tracking analysis, all examined individuals had evidence of the disruption of microbial strain stability. These results are consistent with a previous study using 16S rRNA gene analysis that also found a short-term effect of metformin on the structure of the gut microbial community [18]. In a separate data set of individuals 2 and 4 months after initiation of metformin, we provided evidence for the recovery of the pre-treatment strains by 4 months. One of the features of the use of metformin is the tendency to escalate the dose of the drug over time that is done to allow the patient to accommodate the side effects of diarrhea and flatulence. From our strain-tracking analysis, we found the extent of the recovery of the pre-strains varied between individuals that might correlate with the capacity for the individual to better tolerate the increased dose of metformin. Alternatively, increasing the dose of metformin, which is intended to improve the glucose metabolism, might result in a physiological condition in the GI tract to favor the re-emergence of the minor (non-dominant) microbial strains.

We also examined the impact of two drugs (MTX and T2) with known anti-rheumatic activity for effects on the gut microbial strains. For the treatment with T2, *Faecalibacterium spp*. had the most strain changes followed by the *Bacteroides spp*., while the individuals treated with MTX *Bacteroides spp*. had the most strain changes followed by *Faecalibacterium spp*. MTX has been shown directly to have anti-commensal microbial activity in *in vitro* assays that could account for the strain change [50, 51]. T2 to our knowledge does not have anti-microbial activity. However, T2 does target the expression of heat shock protein and it is possible that the impact of T2 on the microbiome could be through the disruption of the host cells in the GI tract [52].

The disruption of the dominant strain stability in the gut microbial ecosystem by the oral drugs used in our study probably reflects a transient selection. Over time, the minor strain advantage in some individuals is lost resulting in the re-establishment of the dominant (*i*.*e*. pre-drug) strains. The realization that there are individual specific variations in the time to recovery of the dominant strains might have consequences for the health of the host given the function of the microbes in metabolism and protection against invading pathogens [49, 53–56]. We think that it is unlikely that the results of our study are unique to metformin, methotrexate or T2 since several other medications have been found to impact the composition of the gut microbial community [15]. Collectively, our studies then support the concept of monitoring of the status of the gut microbial ecosystem following the initiation of new oral drugs as an important component for evaluating the long-term health of the individual.

## Supporting information

S1 Fig StrainPhlAn on Fukuyama et al. data set. Strain profiling was performed for *Bacteroides vulgatus* using StrainPhlAn across all samples from Fukuyama et al data set. A neighbor-joining (NJ) tree was constructed and the tree is drawn to scale with branch lengths. The distances were computed using the Maximum Composite Likelihood method and are in the units of the number of base substitutions per site using MEGA X. The shaded color boxes shown within the tree match the result found using WSS analysis.
(TIFF)

S2 Fig StrainPhlAn on Sun et al. data set. Strain profiling was conducted for *Bacteroides vulgatus* using StrainPhlAn across all samples from Sun et al data set. A neighbor-joining (NJ) tree was built and the tree is drawn to scale with branch lengths. The distances were measured using the Maximum Composite Likelihood method and are in the units of the number of base substitutions per site using MEGA X. The shaded color boxes shown within the tree match the result found using WSS analysis.
(TIFF)

S3 Fig StrainPhlAn on Wu et al. data set. Strain profiling was conducted for *Bacteroides vulgatus* using StrainPhlAn across all (A) placebo treatment samples and (B) metformin treatment samples from Wu et al data set. A neighbor-joining (NJ) tree was constructed and the tree is drawn to scale with branch lengths. The distances were calculated using the Maximum Composite Likelihood method and are in the units of the number of base substitutions per site using MEGA X. The shaded color boxes shown within the tree match the result found using WSS analysis.
(TIFF)

S4 Fig StrainPhlAn on Zhang et al. data set. Strain profiling was conducted for *Bacteroides vulgatus* using StrainPhlAn across all samples from Zhang et al data set. A neighbor-joining (NJ) tree was built and the tree is drawn to scale with branch lengths. The distances were measured using the Maximum Composite Likelihood method and are in the units of the number of base substitutions per site using MEGA X. The shaded color boxes shown within the tree match the result found using WSS analysis.
(TIFF)

S1 Table Summarized data set used in this study. The table represents a name of data set used in this study, type of treatment, a total number of participants, and citation for each data set.
(XLSX)

S2 Table Sequence reads information. The original sequence files were sequenced and deposited by (A) Fukuyama et al., (accession number: PRJNA388263), (B) Lloyd-Price et al., (accession number: PRJNA398089), (C) Sun et al., (accession number: PRJNA486795), (D) Wu et al., (accession number: PRJNA361402), and (E) Zhang et al. (accession number: PRJEB6997). The table represents the sequence read count before and after the filtering processes.
(XLSX)

S3 Table WSS results from Fukuyama et al. data set. All pairwise comparisons were conducted for individuals between pre and post bowel evacuation (1–10 days and 21–50 days). The resultant WSS scores are showed as numerical value along with the data bar graphs. The WSS scores that were above the cut-off (CO) values are in red. “CO:NA” = No CO value assigned, thus excluded to distinguish a related strain pair. “NS” = No WSS score observed.
(XLSX)

S4 Table WSS results from Lloyd-Price et al., data set. All pairwise comparisons were performed for individuals between early and later week (ranges from 27–44 weeks). The resultant WSS scores are represented as numerical value along with the data bar graphs. The WSS scores that were above the cut-off (CO) values are in red. “CO:NA” = No CO value assigned, thus excluded to distinguish a related strain pair.
(XLSX)

S5 Table WSS results from Sun et al. data set. All pairwise comparisons were performed for individuals between pre and post 3-day metformin treatment. The resultant WSS scores are represented as numerical value along with the data bar graphs. “CO:NA” = No CO value assigned, thus excluded to distinguish a related strain pair.
(XLSX)

S6 Table WSS results from Wu et al. data set. All pairwise comparisons were conducted for individuals between pre and post metformin or placebo treatment (2- and 4-month). In the participant # column, individuals who treated with metformin were colored in red, and individuals who treated with placebo were colored in green. The resultant WSS scores are showed as numerical value along with the data bar graphs. The WSS scores that were above the cut-off (CO) values are in red. “CO:NA” = No CO value assigned, thus excluded to distinguish a related strain pair. “NS” = No WSS score observed.
(XLSX)

S7 Table WSS results from Zhang et al. data set. All pairwise comparisons were conducted for individuals between pre and post MTX or T2 treatment (ranges from 3 to 16 months). In the participant # column, individuals who treated with MTX were labeled with “(MTX)” and individuals who treated with T2 were labeled with “(T2)”. The resultant WSS scores are showed as numerical value along with the data bar graphs. The WSS scores that were above the cut-off (CO) values are in red. “CO:NA” = No CO value assigned, thus excluded to distinguish a related strain pair. “NA” = Sample was not available to run the analysis.
(XLSX)

S8 Table Strain pairs from Wu et al. data set. WSS analysis was conducted for individuals who were treated with (A) metformin for 3-day (Sun et al.), (B) placebo or (C) metformin up to 4 months (Wu et al.), and a total number of related/unrelated strain pairs found from all compared species were listed in the table. The relative abundance (%) was then calculated based on the total number of pairs and listed in the table.
(XLSX)

S9 Table Unrelated strains found across all data sets used in this study. The table summarized a number of unrelated strains found in each data set used in this study. The number in this table match the number shown in Figs 1 and 3–6 (red shaded boxes). NA = Not Applicable.
(XLSX)

## References

[1] Bäckhed F, Fraser CM, Ringel Y, Sanders ME, Sartor RB, Sherman PM, et al Defining a healthy human gut microbiome: current concepts, future directions, and clinical applications. Cell host & microbe. 2012;12(5):611–22. 23159051 10.1016/j.chom.2012.10.012

[2] Shaw LP, Bassam H, Barnes CP, Walker AS, Klein N, Balloux F. Modelling microbiome recovery after antibiotics using a stability landscape framework. The ISME journal. 2019;13(7):1845–56. 10.1038/s41396-019-0392-1 30877283 PMC6591120

[3] Becattini S, Taur Y, Pamer EG. Antibiotic-induced changes in the intestinal microbiota and disease. Trends in molecular medicine. 2016;22(6):458–78. 10.1016/j.molmed.2016.04.003 27178527 PMC4885777

[4] Dethlefsen L, Huse S, Sogin ML, Relman DA. The pervasive effects of an antibiotic on the human gut microbiota, as revealed by deep 16S rRNA sequencing. PLoS biology. 2008;6(11). 10.1371/journal.pbio.0060280 19018661 PMC2586385

[5] Dethlefsen L, Relman DA. Incomplete recovery and individualized responses of the human distal gut microbiota to repeated antibiotic perturbation. Proceedings of the National Academy of Sciences. 2011;108(Supplement 1):4554–61. 10.1073/pnas.1000087107 20847294 PMC3063582

[6] Jernberg C, Löfmark S, Edlund C, Jansson JK. Long-term ecological impacts of antibiotic administration on the human intestinal microbiota. The ISME journal. 2007;1(1):56–66. 10.1038/ismej.2007.3 18043614

[7] Palleja A, Mikkelsen KH, Forslund SK, Kashani A, Allin KH, Nielsen T, et al Recovery of gut microbiota of healthy adults following antibiotic exposure. Nature microbiology. 2018;3(11):1255–65. 10.1038/s41564-018-0257-9 30349083

[8] Raymond F, Ouameur AA, Déraspe M, Iqbal N, Gingras H, Dridi B, et al The initial state of the human gut microbiome determines its reshaping by antibiotics. The ISME journal. 2016;10(3):707–20. 10.1038/ismej.2015.148 26359913 PMC4817689

[9] David LA, Weil A, Ryan ET, Calderwood SB, Harris JB, Chowdhury F, et al Gut microbial succession follows acute secretory diarrhea in humans. mBio. 2015;6(3):e00381–15. 10.1128/mBio.00381-15 25991682 PMC4442136

[10] Fukuyama J, Rumker L, Sankaran K, Jeganathan P, Dethlefsen L, Relman DA, et al Multidomain analyses of a longitudinal human microbiome intestinal cleanout perturbation experiment. PLoS computational biology. 2017;13(8):e1005706 10.1371/journal.pcbi.1005706 28821012 PMC5576755

[11] Gorkiewicz G, Thallinger GG, Trajanoski S, Lackner S, Stocker G, Hinterleitner T, et al Alterations in the colonic microbiota in response to osmotic diarrhea. PLoS One. 2013;8(2). 10.1371/journal.pone.0055817 23409050 PMC3568139

[12] Hsiao A, Ahmed AS, Subramanian S, Griffin NW, Drewry LL, Petri WA, et al Members of the human gut microbiota involved in recovery from Vibrio cholerae infection. Nature. 2014;515(7527):423–6. 10.1038/nature13738 25231861 PMC4353411

[13] Jalanka J, Salonen A, Salojärvi J, Ritari J, Immonen O, Marciani L, et al Effects of bowel cleansing on the intestinal microbiota. Gut. 2015;64(10):1562–8. 10.1136/gutjnl-2014-307240 25527456

[14] O’Brien CL, Allison GE, Grimpen F, Pavli P. Impact of colonoscopy bowel preparation on intestinal microbiota. PloS one. 2013;8(5). 10.1371/journal.pone.0062815 23650530 PMC3641102

[15] Imhann F, Vich Vila A, Bonder MJ, Lopez Manosalva AG, Koonen DP, Fu J, et al The influence of proton pump inhibitors and other commonly used medication on the gut microbiota. Gut Microbes. 2017;8(4):351–8. 10.1080/19490976.2017.1284732 28118083 PMC5570416

[16] Bonnet F, Scheen A. Understanding and overcoming metformin gastrointestinal intolerance. Diabetes, Obesity and Metabolism. 2017;19(4):473–81. 10.1111/dom.12854 27987248

[17] Brunkwall L, Orho-Melander M. The gut microbiome as a target for prevention and treatment of hyperglycaemia in type 2 diabetes: from current human evidence to future possibilities. Diabetologia. 2017;60(6):943–51. 10.1007/s00125-017-4278-3 28434033 PMC5423958

[18] Elbere I, Kalnina I, Silamikelis I, Konrade I, Zaharenko L, Sekace K, et al Association of metformin administration with gut microbiome dysbiosis in healthy volunteers. PloS one. 2018;13(9). 10.1371/journal.pone.0204317 30261008 PMC6160085

[19] Forslund K, Hildebrand F, Nielsen T, Falony G, Le Chatelier E, Sunagawa S, et al Disentangling type 2 diabetes and metformin treatment signatures in the human gut microbiota. Nature. 2015;528(7581):262–6. 10.1038/nature15766 26633628 PMC4681099

[20] Qin J, Li Y, Cai Z, Li S, Zhu J, Zhang F, et al A metagenome-wide association study of gut microbiota in type 2 diabetes. Nature. 2012;490(7418):55–60. 10.1038/nature11450 23023125

[21] Sun L, Xie C, Wang G, Wu Y, Wu Q, Wang X, et al Gut microbiota and intestinal FXR mediate the clinical benefits of metformin. Nature medicine. 2018;24(12):1919–29. 10.1038/s41591-018-0222-4 30397356 PMC6479226

[22] Wu G, Chen J, Hoffman C. Linking long-term dietrary patterns with gut microbial enterotypes. Science. 2011;334(6052):105–8. 10.1126/science.1208344 PubMed Central PMCID: PMC3368382. 21885731 PMC3368382

[23] Kishikawa T, Maeda Y, Nii T, Motooka D, Matsumoto Y, Matsushita M, et al Metagenome-wide association study of gut microbiome revealed novel aetiology of rheumatoid arthritis in the Japanese population. Annals of the rheumatic diseases. 2020;79(1):103–11. 10.1136/annrheumdis-2019-215743 31699813 PMC6937407

[24] Wang Q, Xu R. Data-driven multiple-level analysis of gut-microbiome-immune-joint interactions in rheumatoid arthritis. Bmc Genomics. 2019;20(1):124 10.1186/s12864-019-5510-y 30744546 PMC6371598

[25] Lv Q, Zhang W, Shi Q, Zheng W, Li X, Chen H, et al Comparison of Tripterygium wilfordii Hook F with methotrexate in the treatment of active rheumatoid arthritis (TRIFRA): a randomised, controlled clinical trial. Annals of the rheumatic diseases. 2015;74(6):1078–86. 10.1136/annrheumdis-2013-204807 24733191

[26] Zhang X, Zhang D, Jia H, Feng Q, Wang D, Liang D, et al The oral and gut microbiomes are perturbed in rheumatoid arthritis and partly normalized after treatment. Nature medicine. 2015;21(8):895 10.1038/nm.3914 26214836

[27] Cho I, Blaser MJ. The human microbiome: at the interface of health and disease. Nature Reviews Genetics. 2012;13(4):260–70. 10.1038/nrg3182 22411464 PMC3418802

[28] Kumar R, Yi N, Zhi D, Eipers P, Goldsmith KT, Dixon P, et al Identification of donor microbe species that colonize and persist long term in the recipient after fecal transplant for recurrent Clostridium difficile. NPJ biofilms and microbiomes. 2017;3(1):1–4. 10.1038/s41522-017-0020-7 28649413 PMC5462795

[29] Segata N. On the road to strain-resolved comparative metagenomics. MSystems. 2018;3(2):e00190–17. 10.1128/mSystems.00190-17 29556534 PMC5850074

[30] Truong DT, Tett A, Pasolli E, Huttenhower C, Segata N. Microbial strain-level population structure and genetic diversity from metagenomes. Genome research. 2017;27(4):626–38. 10.1101/gr.216242.116 28167665 PMC5378180

[31] Franzosa EA, Huang K, Meadow JF, Gevers D, Lemon KP, Bohannan BJ, et al Identifying personal microbiomes using metagenomic codes. Proceedings of the National Academy of Sciences. 2015;112(22):E2930–E8. 10.1073/pnas.1423854112 25964341 PMC4460507

[32] Schloissnig S, Arumugam M, Sunagawa S, Mitreva M, Tap J, Zhu A, et al Genomic variation landscape of the human gut microbiome. Nature. 2013;493(7430):45–50. 10.1038/nature11711 PubMed Central PMCID: PMC3536929. 23222524 PMC3536929

[33] Kumar R, Grams J, Chu DI, Crossman DK, Stahl R, Eipers P, et al New microbe genomic variants in patients fecal community following surgical disruption of the upper human gastrointestinal tract. Human Microbiome Journal. 2018;10:37–42. 10.1016/j.humic.2018.10.002

[34] Koo H, Hakim JA, Crossman DK, Kumar R, Lefkowitz EJ, Morrow CD. Individualized recovery of gut microbial strains post antibiotics. NPJ Biofilms Microbiomes. 2019;5:30 Epub 2019/10/22. 10.1038/s41522-019-0103-8 PubMed 31632686 PMC6789009

[35] Koo H, Hakim JA, Crossman DK, Lefkowitz EJ, Morrow CD. Sharing of gut microbial strains between selected individual sets of twins cohabitating for decades. PLOS One. 2019;14(12):e0226111 10.1371/journal.pone.0226111 31805145 PMC6894816

[36] Koo H, McFarland BC, Hakim JA, Crossman DK, Crowley MR, Rodriguez JM, et al An individualized mosaic of maternal microbial strains is transmitted to the infant gut microbial community. Royal Society Open Science. 2020;7:192200 10.1098/rsos.192200 32431894 PMC7211887

[37] Lloyd-Price J, Arze C, Ananthakrishnan AN, Schirmer M, Avila-Pacheco J, Poon TW, et al Multi-omics of the gut microbial ecosystem in inflammatory bowel diseases. Nature. 2019;569(7758):655–62. 10.1038/s41586-019-1237-9 31142855 PMC6650278

[38] Wu H, Esteve E, Tremaroli V, Khan MT, Caesar R, Mannerås-Holm L, et al Metformin alters the gut microbiome of individuals with treatment-naive type 2 diabetes, contributing to the therapeutic effects of the drug. Nature medicine. 2017;23(7):850 10.1038/nm.4345 28530702

[39] Bolger AM, Lohse M, Usadel B. Trimmomatic: a flexible trimmer for Illumina sequence data. Bioinformatics. 2014;30(15):2114–20. 10.1093/bioinformatics/btu170 24695404 PMC4103590

[40] Langmead B, Salzberg SL. Fast gapped-read alignment with Bowtie 2. Nature methods. 2012;9(4):357 10.1038/nmeth.1923 22388286 PMC3322381

[41] Li H, Durbin R. Fast and accurate long-read alignment with Burrows–Wheeler transform. Bioinformatics. 2010;26(5):589–95. 10.1093/bioinformatics/btp698 20080505 PMC2828108

[42] Van der Auwera GA, Carneiro MO, Hartl C, Poplin R, Del Angel G, Levy-Moonshine A, et al From FastQ data to high confidence variant calls: the Genome Analysis Toolkit best practices pipeline. Curr Protoc Bioinformatics. 2013;43:11.0.1–33. Epub 2014/11/29. 10.1002/0471250953.bi1110s43 PubMed 25431634 PMC4243306

[43] Team RC. R: A language and environment for statistical computing [Computer software manual]. Vienna, Austria. 2016.

[44] Segata N, Waldron L, Ballarini A, Narasimhan V, Jousson O, Huttenhower C. Metagenomic microbial community profiling using unique clade-specific marker genes. Nature methods. 2012;9(8):811 10.1038/nmeth.2066 22688413 PMC3443552

[45] Truong DT, Franzosa EA, Tickle TL, Scholz M, Weingart G, Pasolli E, et al MetaPhlAn2 for enhanced metagenomic taxonomic profiling. Nature methods. 2015;12(10):902–3. 10.1038/nmeth.3589 26418763

[46] Tamura K, Nei M, Kumar S. Prospects for inferring very large phylogenies by using the neighbor-joining method. Proceedings of the National Academy of Sciences. 2004;101(30):11030–5. 10.1073/pnas.0404206101 15258291 PMC491989

[47] Stecher G, Tamura K, Kumar S. Molecular evolutionary genetics analysis (MEGA) for macOS. Molecular Biology and Evolution. 2020;37(4):1237–9. 10.1093/molbev/msz312 31904846 PMC7086165

[48] Kumar S, Stecher G, Li M, Knyaz C, Tamura K. MEGA X: molecular evolutionary genetics analysis across computing platforms. Molecular biology and evolution. 2018;35(6):1547–9. 10.1093/molbev/msy096 29722887 PMC5967553

[49] Donaldson GP, Lee SM, Mazmanian SK. Gut biogeography of the bacterial microbiota. Nature Reviews Microbiology. 2016;14(1):20–32. 10.1038/nrmicro3552 26499895 PMC4837114

[50] Bodet CA, Jorgensen J, Drutz D. Antibacterial activities of antineoplastic agents. Antimicrobial agents and chemotherapy. 1985;28(3):437–9. 10.1128/aac.28.3.437 2416271 PMC180269

[51] Maier L, Pruteanu M, Kuhn M, Zeller G, Telzerow A, Anderson EE, et al Extensive impact of non-antibiotic drugs on human gut bacteria. Nature. 2018;555(7698):623–8. 10.1038/nature25979 29555994 PMC6108420

[52] Hingorani SR, Potter JD. Pancreas cancer meets the thunder god. Science translational medicine. 2012;4(156):156ps21–ps21. 10.1126/scitranslmed.3004956 23076355

[53] Bäumler AJ, Sperandio V. Interactions between the microbiota and pathogenic bacteria in the gut. Nature. 2016;535(7610):85–93. 10.1038/nature18849 27383983 PMC5114849

[54] Buffie CG, Pamer EG. Microbiota-mediated colonization resistance against intestinal pathogens. Nature Reviews Immunology. 2013;13(11):790–801. 10.1038/nri3535 Epub 2013 Oct 7. 24096337 PMC4194195

[55] Lloyd-Price J, Abu-Ali G, Huttenhower C. The healthy human microbiome. Genome medicine. 2016;8(1):1–11. 10.1186/s13073-015-0257-9 27122046 PMC4848870

[56] Maynard CL, Elson CO, Hatton RD, Weaver CT. Reciprocal interactions of the intestinal microbiota and immune system. Nature. 2012;489(7415):231 10.1038/nature11551 22972296 PMC4492337

