# Supplementary figures and images for: Perturbation of the human gastrointestinal tract microbial ecosystem by oral drugs to treat chronic disease results in a spectrum of individual specific patterns of extinction and persistence of dominant microbial strains
Source: PLoS One. 2020 Dec 1;15(12):e0242021. doi: 10.1371/journal.pone.0242021 (PMC7707550; doi:10.1371/journal.pone.0242021)

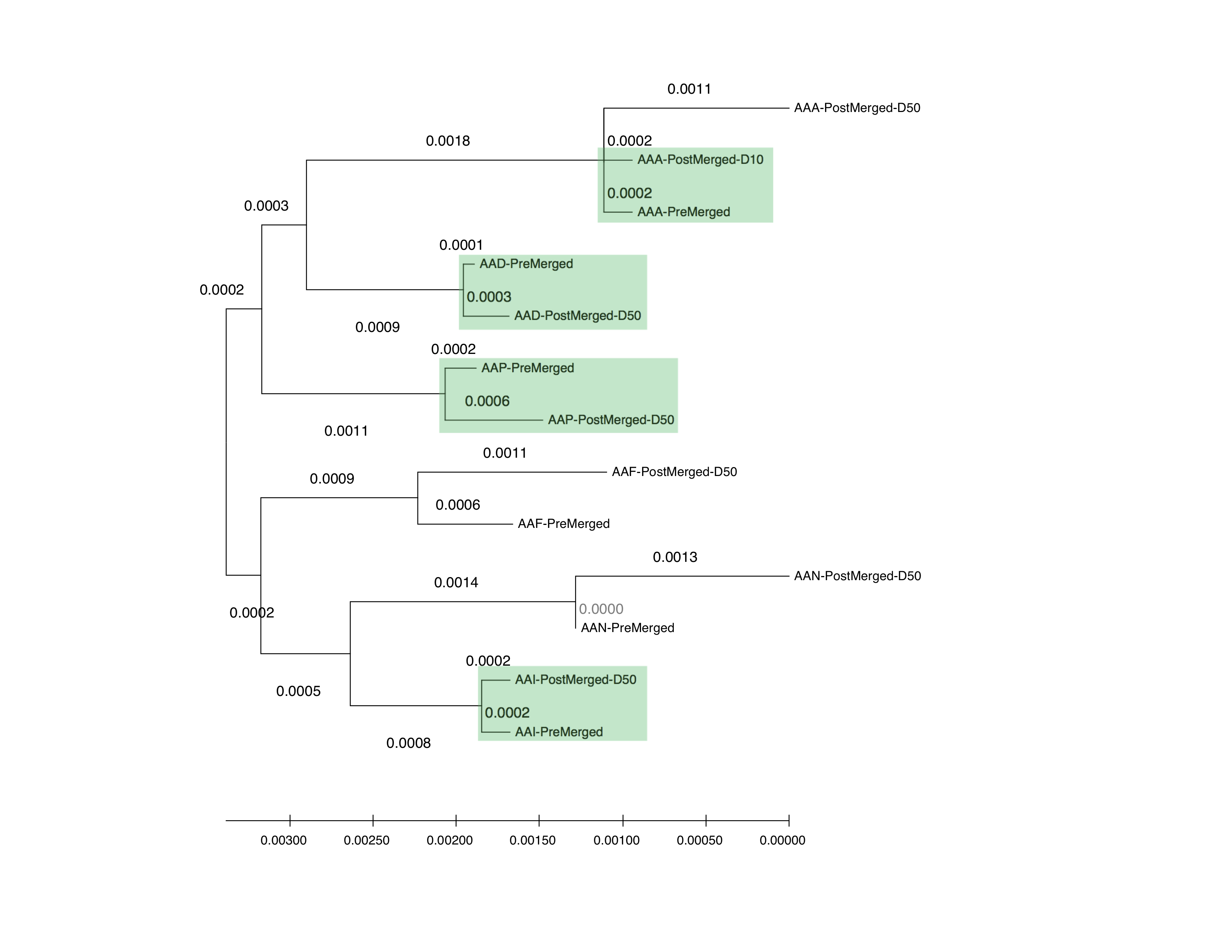

Supplement: S1 Fig — Strain profiling was performed for Bacteroides vulgatus using StrainPhlAn across all samples from Fukuyama et al data set. A neighbor-joining (NJ) tree was constructed and the tree is drawn to scale with branch lengths. The distances were computed using the Maximum Composite Likelihood method and are in the units of the number of base substitutions per site using MEGA X. The shaded color boxes shown within the tree match the result found using WSS analysis. (TIFF) [file pone.0242021.s001.tiff]

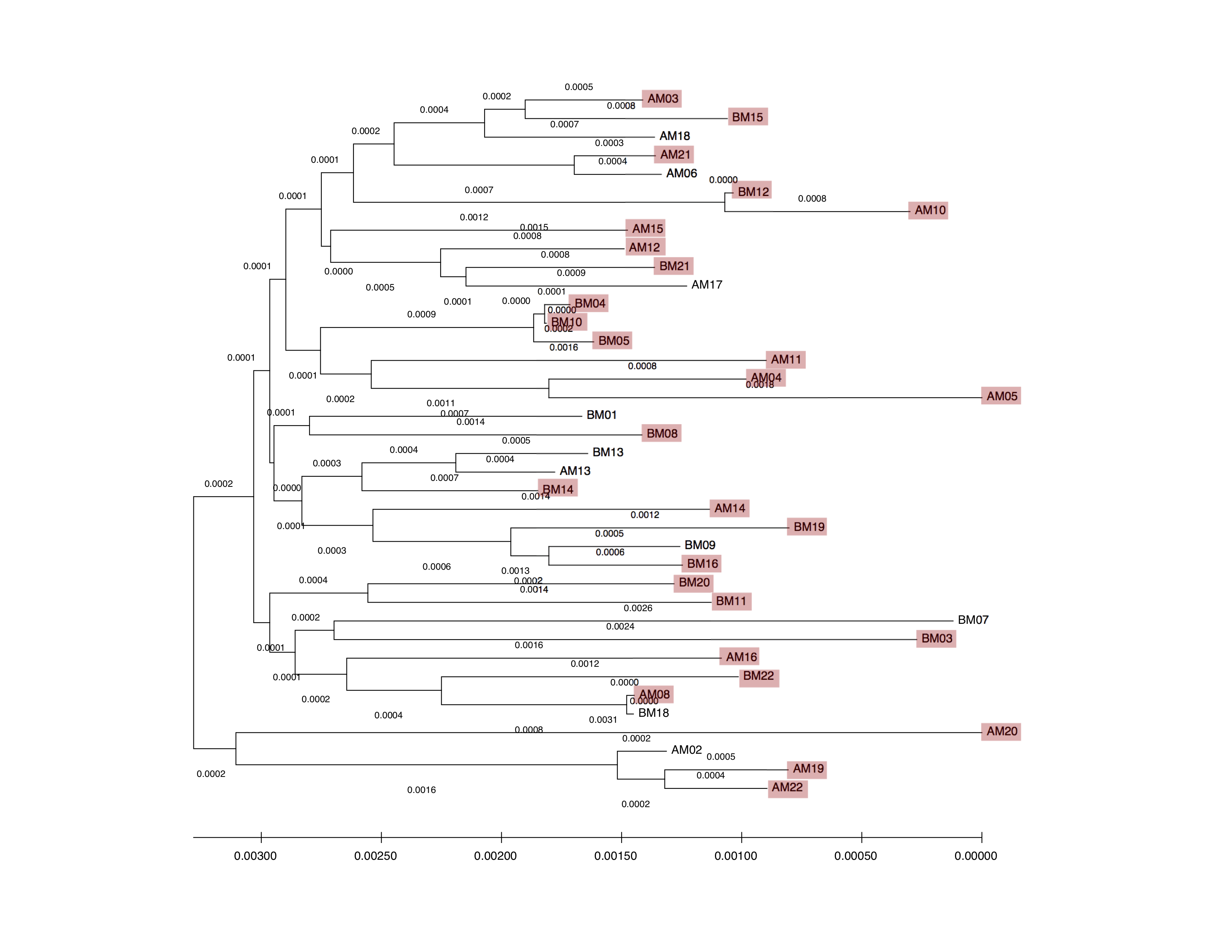

Supplement: S2 Fig — Strain profiling was conducted for Bacteroides vulgatus using StrainPhlAn across all samples from Sun et al data set. A neighbor-joining (NJ) tree was built and the tree is drawn to scale with branch lengths. The distances were measured using the Maximum Composite Likelihood method and are in the units of the number of base substitutions per site using MEGA X. The shaded color boxes shown within the tree match the result found using WSS analysis. (TIFF) [file pone.0242021.s002.tiff]

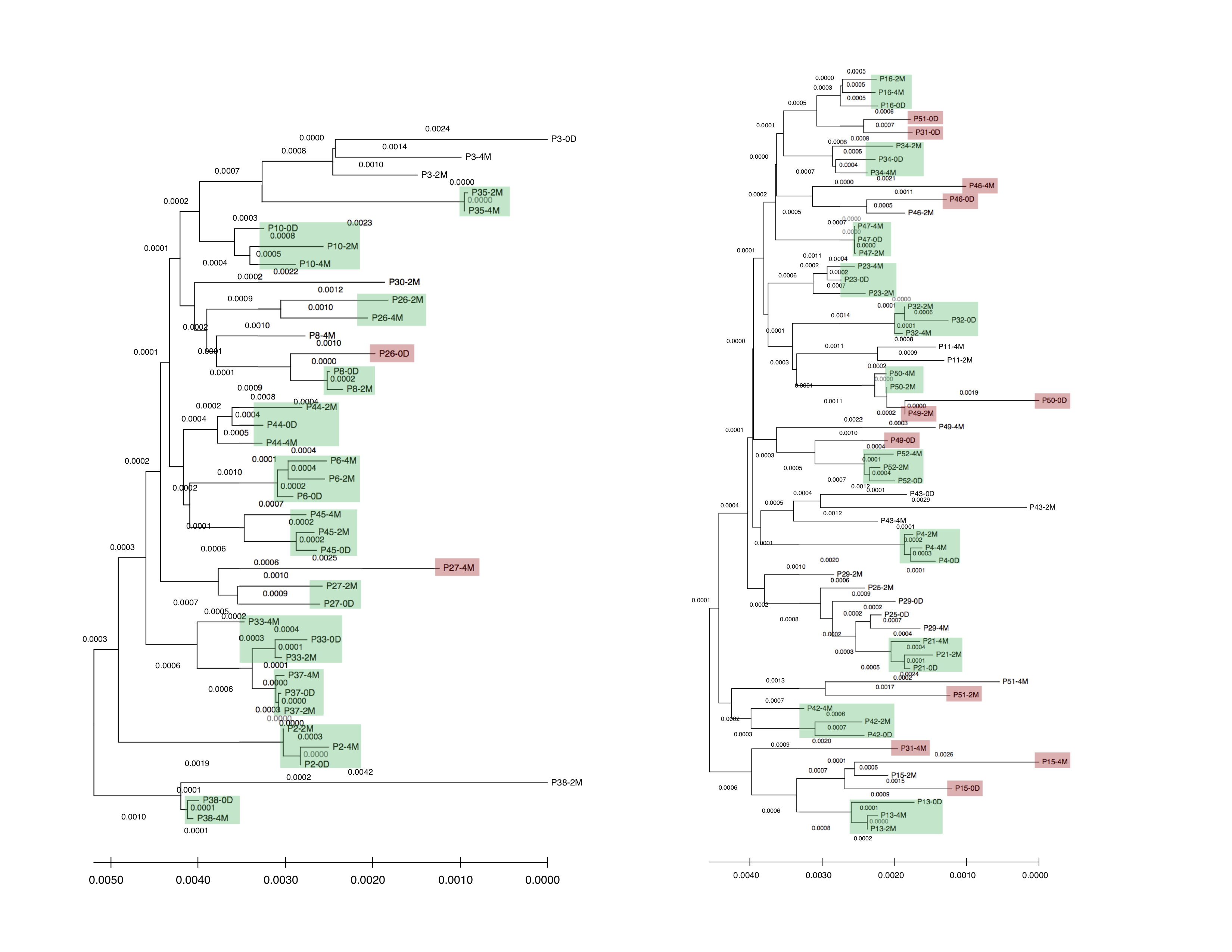

Supplement: S3 Fig — Strain profiling was conducted for Bacteroides vulgatus using StrainPhlAn across all (A) placebo treatment samples and (B) metformin treatment samples from Wu et al data set. A neighbor-joining (NJ) tree was constructed and the tree is drawn to scale with branch lengths. The distances were calculated using the Maximum Composite Likelihood method and are in the units of the number of base substitutions per site using MEGA X. The shaded color boxes shown within the tree match the result found using WSS analysis. (TIFF) [file pone.0242021.s003.tiff]

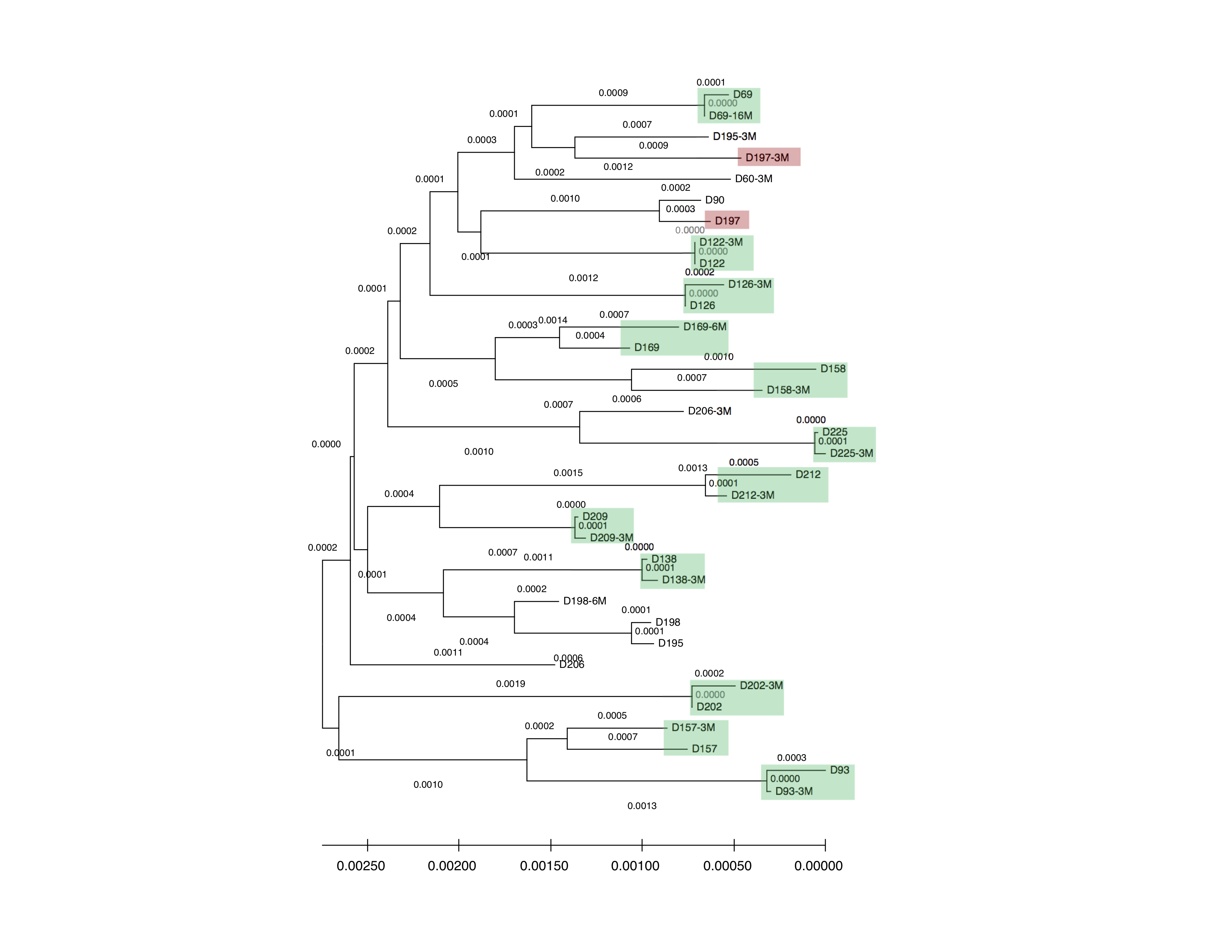

Supplement: S4 Fig — Strain profiling was conducted for Bacteroides vulgatus using StrainPhlAn across all samples from Zhang et al data set. A neighbor-joining (NJ) tree was built and the tree is drawn to scale with branch lengths. The distances were measured using the Maximum Composite Likelihood method and are in the units of the number of base substitutions per site using MEGA X. The shaded color boxes shown within the tree match the result found using WSS analysis. (TIFF) [file pone.0242021.s004.tiff]
